# Hydrogen-Bonding Changes Cause Differences in Imipenem Breakdown Activity in OXA-48 Variants

**DOI:** 10.1021/acs.jcim.6c00223

**Published:** 2026-04-22

**Authors:** Daojiong Wang, Adrian J. Mulholland, James Spencer, Marc W. van der Kamp

**Affiliations:** a School of Biochemistry and Cellular and Molecular Medicine, 1980 University of Bristol, Bristol BS8 1TD, England; b Centre for Computational Chemistry, School of Chemistry, 1980 University of Bristol, Bristol BS8 1TS, England

## Abstract

The β-lactamase OXA-48 efficiently hydrolyzes carbapenem antibiotics, especially imipenem. Carbapenem resistance is a rising clinical concern and is frequently associated with OXA-48 and its variants. OXA-48 variants carrying different mutations in the β_5_–β_6_ loop differ in hydrolytic activity toward imipenem. OXA-517 has a higher *K*_M_, but a similar *k*_cat_ for imipenem hydrolysis, compared to that of OXA-48, whereas those of OXA-163 and -405, which have similar mutations in the β_5_–β_6_ loop, are less active. Multiscale simulations (using quantum mechanics/molecular mechanics, QM/MM) of deacylation of the respective imipenem acylenzymes show this to be most efficient when the deacylating water (DW) acts as a hydrogen bond (H-bond) donor to imipenem, and the carboxylated Lys73 base is less hydrated. Calculated barriers for deacylation correlate very well with experimental data but, for OXA-163 and -405, only when DW acts as an H-bond acceptor. Molecular dynamics simulations of imipenem acylenzyme complexes show that mutations in the β_5_–β_6_ loop change the active site H-bond network. In OXA-48, the DW H-bonding pattern linked to high activity is more frequently sampled, and in OXA-517, it is stabilized through H-bonding to Thr213, explaining the higher *k*_cat_ values compared to those of OXA-163 and -405, where this is not the case. Furthermore, simulations of noncovalent imipenem complexes indicate that increased *K*_M_ for OXA-517 is linked to lower binding affinity caused by the repositioning of bound imipenem. Our work identified the molecular basis for differences in imipenem hydrolytic activity between OXA-48 variants, offering detailed insights into how active site interactions alter the dynamics and reaction efficiencies related to antibiotic resistance.

## Graphical abstract

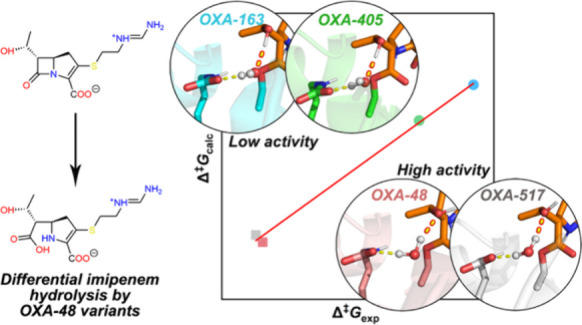

## Introduction

Antimicrobial resistance (AMR) has become a serious threat to public health.^1,2^ The discovery of antibiotics is one of the most important findings in the 20th century. However, under the pressure to survive, bacteria have evolved or acquired various ways to protect themselves against antimicrobial agents, leading to 1.14 million deaths in 2021 directly attributable to antimicrobial resistance.^3^ One of the major resistance mechanisms in bacteria is the production of β-lactamases, enzymes that hydrolyze β-lactam antibiotics, the most widely used treatment for bacterial infections worldwide.^4,5^

In β-lactam antibiotics, the β-lactam ring mimics the d-Ala-d-Ala moiety of the natural substrate of penicillin-binding proteins (PBPs), a family of enzymes essential to maintaining bacterial cell wall structure and stability.^6,7^ Bacteria can produce enzymes called β-lactamases (BLs) that hydrolyze the β-lactam ring, thus leading to antibiotic inactivation.^8^ BLs are divided into four classes, A, C, D and B, based on the Ambler (sequence-based) classification^9^ system: class B BLs are zinc-dependent, whereas classes A, C, and D are serine BLs that all have an active site serine residue that performs a nucleophilic attack on the β-lactam ring and so are mechanistically related to the transpeptidase activity of PBPs.^10,11^ The OXA-48-like family of class D β-lactamases is particularly relevant for antibiotic resistance due to their wide distribution^12^ and the fact that many members of the family have significant hydrolytic activity against carbapenems, which used to be considered “last-resort” antibiotics.^13^ The OXA-48 parent enzyme has a higher hydrolytic activity toward imipenem than other carbapenems.^14^ The presence of OXA-48-like enzymes, in combination with other resistance mechanisms, can lead to high levels of carbapenem resistance.^13,15^

The general mechanism of imipenem hydrolysis by OXA-48-like enzymes consists of three main stages: initial noncovalent binding (Michaelis complex formation), acylation, and deacylation (Figure 1).^14^ Both acylation and deacylation involve a general base. Unlike class A BLs, which are expected to utilize Glu166 as the general base in both acylation and deacylation,^16–18^ class D BLs such as the OXA-48-like enzymes employ a carboxylated Lys73 for both.^19,20^ After the formation of the noncovalent Michaelis complex, acylation occurs, in which the carboxylated Lys73 (KCX) functions as a general base to activate Ser70.^19^ Ser70 then performs a nucleophilic attack upon the carbonyl carbon of the β-lactam ring, opening the ring and forming a covalent bond with the electrophilic carbon. The resulting acylenzyme complex (AC) is deacylated to release the product and let the enzyme turn over: this step was determined to be the rate-limiting step in imipenem hydrolysis by OXA-48 and -163 (through pre-steady-state kinetic analysis).^21^ Deacylation involves an active site water molecule, known as deacylating water (DW), which is activated by KCX to act as a nucleophile. This nucleophilic attack on the carbonyl carbon leads to a short-lived tetrahedral intermediate (TI), with the energy barrier associated with this step explaining the difference between OXA-48 activity toward imipenem and meropenem,^22^ further suggesting that the TI formation in deacylation is rate-determining. The second step of deacylation involves bond breakage between Ser70 and hydrolyzed imipenem followed by release of the final product. The expectation is that the imipenem pyrroline ring is in its Δ^2^ (enamine) tautomeric form to allow for efficient deacylation, as was confirmed for the class A carbapenemase KPC-2.^23^ As well as the Δ^2^ tautomer, both stereoisomers of the alternative Δ^1^ tautomer (*R*-Δ^1^ and *S*-Δ^1^, imine) have been observed in OXA-48 acylenzyme crystal structures.^21,24–26^ However, the Δ^1^ tautomer may inhibit the enzyme,^27^ consistent with NMR studies that indicate that OXA-48 and other carbapenemases form a Δ^2^ or an *R*-Δ^1^ deacylation product (with the latter potentially occurring through rapid nonenzymatic tautomerization).^28^

**Figure 1:**
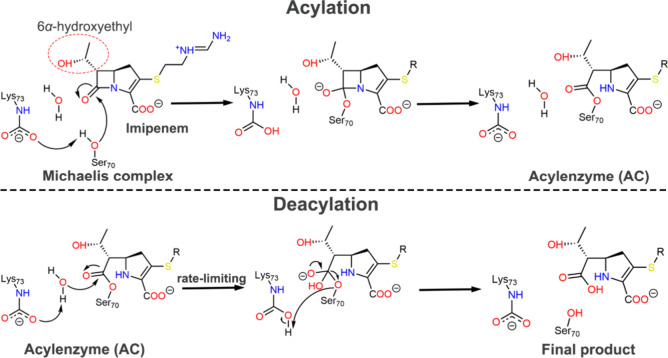
Reaction mechanism of imipenem hydrolysis (via its Δ^2^ tautomer) by OXA-48-like enzymes.^10,14^ Carboxylation of Lys73 is required for efficient hydrolysis.^19,20^

OXA-48-like enzymes (as well as most other class D BLs) have three widely conserved active site motifs.^14,20^ Motif I (S^70^-X-X-K^73^) contains Ser70 and Lys73, which can be carboxylated, both of which are essential for the reaction (see above). Although motif II (S^118^-V-V) and motif III (K^208^-T-G) are distant from motif I in sequence, they are located close to the key catalytic residues (on a helix and a beta-strand, respectively). Val120 forms a “water channel” with Leu158, allowing a water molecule (DW) to enter the active site and support deacylation.^24,29^ Val120 also provides a hydrophobic environment that lowers the p*K*_a_ of Lys73, thereby facilitating its carboxylation.^30,31^ Lys208 from motif III forms a hydrogen bond to the side chain of Ser118 and has been proposed to be involved in substrate recognition.^31^ Alanine substitution at Ser118 and Lys208 was reported to cause a significant reduction in MICs for carbapenems.^32^ The Ω loop (residues Tyr144 to Arg163 in OXA-48) and β_5_–β_6_ loop (residues Thr213 to Lys218 in OXA-48) are not directly involved in the reaction, but these loops have been indicated to play important roles in enzyme specificity. The enhanced flexibility of the Ω and β_5_–β_6_ loops in lab-evolved OXA-48 variants (compared to the parent OXA-48) has been suggested to cause increased efficiency toward ceftazidime.^33–35^

Several natural, clinically relevant variants of OXA-48 have changes in the β_5_–β_6_ loop. OXA-163, a commonly encountered OXA-48 variant, contains a four-residue deletion (214-RIEP-217) and single residue substitution (S212D). OXA-163 possesses significant cephalosporin hydrolysis activity,^36^ whereas carbapenem hydrolysis efficiency, particularly for imipenem, is significantly reduced compared to OXA-48 (Figure 2).^21,37^ It was hypothesized, based on single MD trajectories of substrate complexes, that this reduction is related to multiple conformations or substates being sampled by OXA-163 (compared to OXA-48).^21^ Another variant, OXA-405, has a similar four-residue deletion (213-TRIE-216), leading to similar effects on the hydrolytic activity. The replacement of the β_5_–β_6_ loop of OXA-48 with that of OXA-18, which can hydrolyze cephalosporins, also results in the ability to hydrolyze cephalosporins.^38^ In both of these variants, Arg214 in the β_5_–β_6_ loop, which can form a salt bridge interaction with Asp159 to help stabilize the Ω loop in OXA-48 resulting in a higher melting point of the protein, is missing.^39^ Mutation studies of Arg214 in OXA-48 and OXA-232 suggest that this residue is crucial for carbapenemase activity by OXA-48-like enzymes.^40^

**Figure 2:**
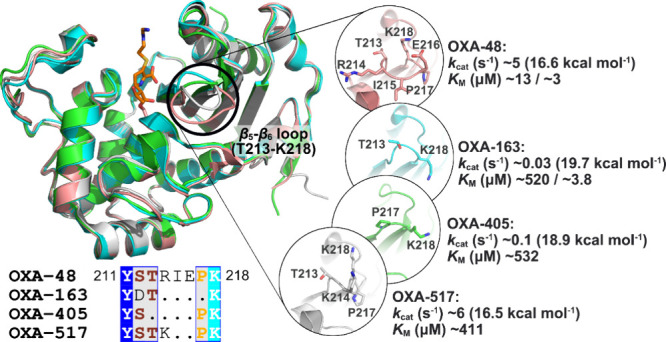
Aligned crystal structures of OXA-48 (pink, PDB ID: 6P97,^24^ chain A, imipenem acylenzyme complex), OXA-163 (cyan, PDB ID: 7KHZ,^21^ chain A, imipenem acylenzyme complex), OXA-405 (green, PDB ID: 5FDH,^42^ chain A, apo), and OXA-517 (gray, PDB ID: 6HB8,^41^ chain A, apo) (upper panel). For clarity, the covalently bound imipenem is shown as sticks (orange carbons) only in OXA-48. The positions of the imipenem core in the peptide in OXA-163 are highly similar. Sequences of the β_5_–β_6_ loop are displayed below (full sequence alignment in Figure S1). Structural differences in the β_5_–β_6_ loops are highlighted in the insets, together with experimental kinetic data obtained from published studies.^20,21,37,41,42^ Different experimental *K*_M_ values of OXA-48 and -163 have been reported, as indicated. Experimental *k*_cat_ values were converted into activation free energies (Δ^‡^ *G*, in kcal mol^–1^) at 300 K using the transition state theory.^43^

Recently, a new OXA-48 variant, OXA-517, was discovered.^41^ OXA-517 harbors a two-residue deletion (215-IE-217) and one residue substitution (R214 K) in the β_5_–β_6_ loop compared to those of OXA-48. Notably, even though OXA-517 has mutations in the β_5_–β_6_ loop, it shares a similar *k*_cat_ value for imipenem hydrolysis with OXA-48 (whereas *k*_cat_ values are much lower for OXA-163 and -405; Figure 2 and Table S1).^20,37,41,42^ However, OXA-517 does have a higher *K*_M_ value than OXA-48, causing a lower hydrolysis efficiency overall. Clearly, the main difference between OXA-48 and the variants mentioned (OXA-163, -405, and -517) is in the β_5_–β_6_ loop (Figure 2and Figure S1), but how mutations in the β_5_–β_6_ loop affect carbapenemase activity remains unclear.

Here, we use detailed atomistic simulations to investigate the differences in imipenem hydrolysis observed for the OXA-48-like enzymes with changes in their β_5_–β_6_ loops. Multiscale (QM/MM) reaction simulations were used to investigate the rate-limiting deacylation step. Our results support that for each variant studied, the energetically most favorable situation for deacylation is a less hydrated catalytic base (KCX), with the DW acting as hydrogen bond donor to the imipenem 6α-hydroxyethyl group, as previously found for OXA-48.^22^ Molecular dynamics simulations and QM/MM calculations of the different acylenzyme complexes revealed a significant difference between the variants in the preference of H-bonding patterns formed around the DW. Whereas the differences in the β_5_–β_6_ loop disfavor the DW acting as hydrogen bond donor in OXA-163 and -405, OXA-517 still allows this interaction, which favors imipenem deacylation. Furthermore, molecular dynamics simulations of the noncovalent imipenem complexes show that in OXA-517, the imipenem position differs from that in OXA-48, leading to lower binding affinity and thus a change in *K*_M_. Overall, our results explain the changes in hydrolytic activity toward imipenem for key variants of OXA-48 with mutations in the β_5_–β_6_ loop, providing information on how subtle conformational differences cause changes in enzyme activity, aiding in a deeper understanding of antibiotic resistance conferred by β-lactamases.

## Methods

### System Setup and Preparation

Simulations applied protocols similar to those used in previous studies.^22,44^ The X-ray crystal structures of imipenem acylenzyme complexes binding with OXA-48 and OXA-163 were obtained from the PDB database (PDB ID: 6P97,^24^ chain A, and PDB ID: 7KHZ,^21^ chain A, respectively). The initial structures of imipenem acylenzyme complexes of OXA-405 and OXA-517 were generated by modeling imipenem into the apo X-ray structures of OXA-405 (PDB ID: 5FDH,^42^ chain A) and OXA-517 (PDB ID: 6HB8,^41^ chain A) through alignment with the OXA-163 and OXA-48 acylenzyme complexes, respectively. The missing atoms of the carboxylated Lys73 were added to the acylenzyme complexes of OXA-48, -405, and -517 acylenzyme complexes, and the deacylating water (DW) was added manually to all. The OXA-163 acylenzyme complex, in which Lys73 is substituted by Ala73 in the crystal structure, was reverted to a carboxylated Lys73. Noncovalent (Michaelis) complexes of imipenem with the enzymes were generated through breaking the bond between Ser70 and imipenem in the previously prepared acylenzyme complexes and reforming the β-lactam ring manually in PyMOL. Partial charges for the acylated imipenem (including Ser70) were calculated by using RESP charge calculation based on HF/6-31G­(d) of the capped Ser70-imipenem fragment using the R.E.D. server.^45^ For the noncovalently bound imipenem, partial charges were determined using the AM1-BCC model using antechamber.^46^ The GAFF2 force field^47^ was used to describe the atom types and bonded parameters of both forms of imipenem. All waters in the crystal structures were retained (but ions and buffer molecules deleted), and the protonation states of ionizable residues at pH 7.0 were determined using PropKa3.1,^48^ with histidine tautomers predicted with the reduce program from AmberTools.^49^ All ionizable residues were predicted to be in their standard protonation states (Asp and Glu deprotonated; Lys and Arg protonated), with His34, His90, His109, His178, and His182 singly protonated on NE2 and His38 and His140 singly protonated on ND1. Protein atoms were treated with the Amber ff14SB force field.^50^ All complexes were solvated in a rectangular box of TIP3P water,^51^ with a minimum distance between the protein and the box edge of 10 Å. To neutralize the complexes, one Na^+^ ion was added to OXA-48 and OXA-405 and two Na^+^ ions were added to OXA-163 by replacing random bulk water molecules (no ions were added to OXA-517).

### MM Molecular Dynamics (MD) Simulation and Analysis

All systems were first minimized for 2000 cycles (1000 cycles with the steepest descent followed by 1000 cycles with a conjugate gradient). Then, the temperature was increased from 50 to 300 K over a period of 20 ps. During all equilibration and production simulations, periodic boundary conditions were applied, and the SHAKE algorithm was applied to fix all bond lengths involving hydrogen atoms. A time step of 2 fs was used, and the cutoff radius for nonbonded interactions was set to 8.0 Å.

For each acylenzyme complex, five independent simulations were run. Initially, unrestrained simulations of 120 ns were run in the NPT ensemble at 300 K (maintained using Langevin dynamics, collision frequency 0.2 ps^–1^) and 1 bar (using Berendsen barostat with isotropic position scaling, pressure relaxation time 1 ps). The carbapenem 6α-hydroxyethyl group can adopt three rotameric states or orientations (according to dihedral angle, C7-C6-C16-O7) (Figure S2),^52,53^ and previous MM MD simulation of the OXA-48 imipenem acylenzyme complex shows that the 6α-hydroxyethyl group can adopt all three.^22^ QM/MM simulation of imipenem (and meropenem) hydrolysis by OXA-48 indicates that the different orientations significantly influence the energy barrier, with orientation I (∼50°) the most reactive rotameric state.^22^ Therefore, this orientation was enforced here. First, one frame per replica was selected from the initial restraint-free production simulations in which the imipenem 6α-hydroxyethyl group adopted the rotamer captured in the OXA-48 and OXA-163 imipenem acylenzyme crystal structures. Then, the imipenem 6α-hydroxyethyl group was gradually changed to ∼50° by applying a dihedral angle restraint (force constant of 100 kcal mol^–1^ rad^–2^) and changing it by 5° every 100 ps (27 steps). The final frame from the last step was used to perform a further 120 ns MM MD simulation with a two-sided harmonic dihedral angle restraint (force constant of 100 kcal mol^–1^ rad^–2^) on the 6α-hydroxyethyl group, allowing restraint-free sampling from 0° to 100°. The first 20 ns of this simulation was regarded as further (postrestraint) equilibration and was excluded from trajectory analysis. The trajectories were saved every 10 ps, resulting in 10,000 frames per replica for analysis and a total of 50,000 frames per system.

To investigate differences in relative binding energy, 10 independent simulations of Michaelis complexes (imipenem noncovalently bound) were conducted of 10.5 ns each (with the first 0.5 ns simulation treated as equilibration). Because imipenem can (partially) dissociate from enzyme in simulation, two weak one-sided harmonic restraints were applied throughout the simulations for the oxyanion hole hydrogen-bond distances to capture the acylation-ready state. The restraints came into force when the distance between the carbonyl oxygen of imipenem and the backbone amide H atom of Ser70/Tyr211 was above 2.0 Å (force constant 10 kcal mol^–1^ Å^–2^). The trajectories were saved every 10 ps, resulting in 1000 frames per replica, and a total of 10000 frames per system were used for analysis.

All simulations were conducted using Amber20/AmberTools20, and trajectories were analyzed using CPPTRAJ.^49,54^ Heavy atom RMSD values of the Ω loop were calculated by aligning the trajectories on all Cα atoms, excluding those in the Ω loop. The first frame of each trajectory was used as reference. Differences in Cα fluctuation (ΔRMSF_Cα_) were calculated by using the RMSF per residue of each variant minus the RMSF value of the corresponding residue in OXA-48. The RMSF values for each residue Cα were determined by first obtaining the average coordinate set of each replica and then aligning the trajectory to this coordinate set prior to RMSF calculation. An independent two-sample, two-tailed *t* test was performed to compare the per-residue RMSF values of the variants of OXA-48 with those of WT OXA-48 (*n* = 5). Clustering analysis was conducted with the k-means algorithm, and the number of clusters was determined based on the highest pseudo-*F* statistic (pSF) value. For clustering of acylenzyme complexes, the trajectories were first aligned to global average structure (residues Arg214-Pro217 in OXA-48 and Lys214-Pro215 in OXA-517 were excluded to ensure positional correspondence across all enzymes), after which clustering was performed based on RMSD of Cα atoms. For clustering of Michaelis complexes, the trajectories were first aligned to the Cα atoms of active site residues that are located within 5 Å of the ligand (residues Ile102-Trp105, Lys116-Val120, Lys208-Ser212, and Leu247-Arg250 in OXA-48). Then, clustering was performed based on the RMSD of heavy atoms of the imipenem core (Figure S3). Principal component analysis was performed after aligning the trajectories to the global average structure. For hydrogen bond analysis, the default criteria in CPPTRAJ were used (a hydrogen donor–acceptor distance less than 3.0 Å and a donor–hydrogen–acceptor angle between 135° and 180°).

### QM/MM MD Reaction Simulation and Analysis

To investigate the reaction barrier of deacylation, a 2D QM/MM umbrella sampling MD was applied to the acylenzyme complexes of imipenem with the OXA-48-like proteins. Frames from the last 40 ns of MM MD simulation were selected based on a reactive DW position (distance between DW@O and KCX@OQ1 closer than 3.0 Å and distance between DW@O and electrophilic carbon closer than 3.5 Å), together with the desired DW H-bonding pattern and hydration state. The core structure of imipenem, side chains of Ser70 and carboxylated Lys73, and the deacylating water are included in the QM region (Figure S3). The QM region comprises 43 atoms (including three link atoms) and carries a total charge of −2 e. The QM region was described by the semiempirical method DFTB2 (SCC-DFTB).^55^ DFTB2 was shown to provide reasonable geometries and to reproduce experimental differences in enzyme kinetics for carbapenem deacylation in OXA-48 in our previous work.^22^ Simulation settings for QM/MM were identical to the previous MM simulations, apart from using a 1 fs time step (and no SHAKE applied on the QM region). Selected frames were used to calculate a free energy surface (FES) by using umbrella sampling (US), applying two reaction coordinates. One is a reaction coordinate following the proton transfer between KCX and DW: *d*[KCX@OQ1, DW@H] – *d*[DW@O, DW@H]) which ensures the break of the O–H bond in DW and formation of the KCX@OQ1-DW@H bond. The other is a nucleophilic attack coordinate, following the OH^−^ of DW approaching the electrophilic carbon (*d*[DW@O, IME@C7]), and forming a covalent bond. From the acylenzyme complex to tetrahedral intermediate, the proton transfer coordinate value changes from 0.8 to −1.0 Å, while for nucleophilic attack, it changes from 3.5 to 1.5 Å (both in steps of 0.1 Å).

Umbrella sampling was first performed window-by-window along the approximate minimum free energy path used in a previous work,^22^ and then the full 2D surface was covered by going outward from this minimum free energy path. A total of 399 US windows were used, with 2 ps MD sampling in each window for each replica. This allows for sufficient sampling to uncover trends while maintaining the specific active site conformation.^22^ To avoid changes in hydrogen bonding of the water surrounding the carboxylated Lys73 during the reaction simulations, distance and angle restraints were applied (see Note S1 for details). Reaction coordinate values were saved every step and analyzed using the weighted histogram analysis method (WHAM).^56^ The Minimum Energy Path Surface Analysis (MEPSA) program^57^ was used to plot the resulting FES and the minimum free energy path.

### QM/MM Potential Energy Calculations for H-Bond Network Changes

To investigate whether the wider H-bond network can support the reaction favored H-bond pattern of the DW (as hydrogen donor), a series of QM/MM minimization and single point energy calculations were conducted. Three representative frames from the acylenzyme MD simulation of each system where the DW acts as a hydrogen acceptor and Thr213 (if present) is involved in the H-bond network were selected. The core structure of imipenem, side chain of Ser70, part of the Tyr211 backbone (cut at the Cα-C bond), whole Ser/Asp212 backbone, and part of Thr213/Pro217 backbone (cut at the Cα-C bond) with the entire side chain, DW, and waters involved in the H-bond network were included in the QM region (described by DFTB2). For OXA-48, -163, and -405, the QM regions contain 60 atoms (including five link atoms), whereas for OXA-517, it contains 63 atoms (including five link atoms) due to an additional water molecule. The total charge of each QM region is −1 e. The whole complex was first minimized using the LBFGS^58^ optimizer, with only atoms within 20 Å of the Cα atom of Ser/Asp212 allowed to move. The convergence criterion for the energy gradient was set to 0.02 kcal mol^–1^ Å^–1^, and no nonbonded cutoff was applied. The dihedral angle (C6-C16-O7-H8) of the imipenem 6α-hydroxyethyl hydroxyl group was restrained to −80° (Figure S2) using a two-sided harmonic dihedral restraint with a force constant of 3000 kcal mol^–1^ rad^–2^. Then, further minimizations were conducted in a stepwise manner, allowing only atoms within 15 Å of the Cα atom of Ser/Asp212 to move (to avoid potential energy changes not related to the changing H-bonding pattern). In each step, the dihedral angle was increased by 10° until a local minimum was reached, at which point the deacylating water acted as hydrogen donor and the H-bond network had adjusted correspondingly. The QM/MM potential energy difference was computed between the two local minima, one corresponding to the deacylating water acting as hydrogen acceptor and the other as hydrogen donor. For accuracy, DFTB2/ff14SB energies were corrected by replacing DFTB2 energies of the QM region only with the corresponding M06-2X/def2-TZVP energies, resulting in M06-2X/def2-TZVP//ff14SB energies (with QM-MM interaction terms still at the DFTB2 level). M06-2X/def2-TZVP calculations were performed in ORCA 6.0 ^59^ with the RIJCOSX approximation and the def2/J auxiliary basis set.

### Binding Energy Calculation (MM/GBSA)

MM/GBSA (with energy decomposition per residue) was performed to calculate the binding energy of imipenem to the different enzyme variants using MMPBSA.py^60^ from AmberTools20. All trajectory snapshots from the Michaelis complex simulations were used. Topology files for MM/GBSA calculations were prepared by using the mbondi2 radii, as recommended with the Onufriev, Bashford, and Case (OBC) generalized Born model used here.^61^ Residues with significant contributions to the binding energy (absolute value of contribution >0.5 kcal mol^–1^) were selected, and a comparison between OXA-48 variants and OXA-48 was constructed.

## Results and Discussion

### Difference in Imipenem Deacylation Rates in OXA-48 Variants Explained by the Different Orientation of the Deacylating Water

Our previous work on carbapenem deacylation by WT OXA-48 showed that the rotamer of the carbapenem 6α-hydroxyethyl group and the hydration state around the carboxylated Lys73 (KCX) are both important factors influencing the free energy barrier.^22^ When the 6α-hydroxyethyl group is in the most reactive conformation (rotamer orientation I, dihedral ∼50°), the DW can form two different H-bonding patterns with the carbapenem 6α-hydroxyethyl hydroxyl group (Figure 3 and Figure S2). Here, we perform QM/MM (DFTB2/ff14SB) umbrella sampling reaction simulations with imipenem in this most reactive orientation to investigate the differences in *k*_cat_ between OXA-48 and its β_5_–β_6_ loop variants OXA-163, OXA-405, and OXA-517. Different H-bonding patterns and hydration states are considered separately (Figure 3).

**Figure 3:**
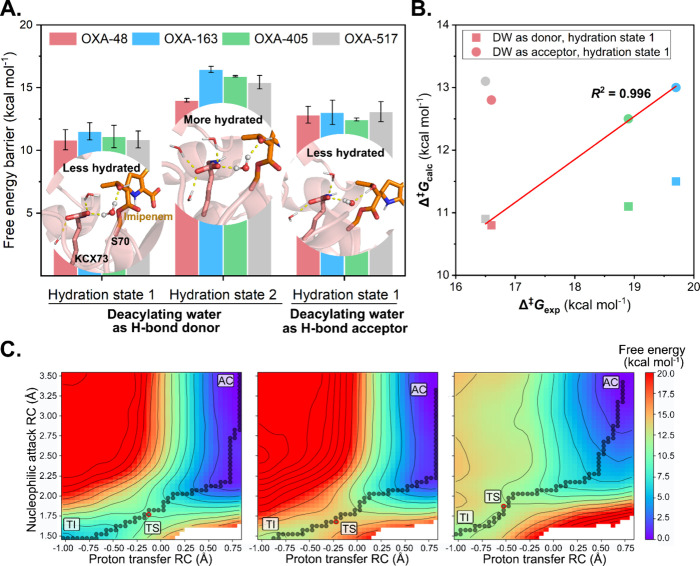
Predicted free energy barriers for deacylation of imipenem acylenzyme complexes of OXA-48 variants in different H-bonding patterns and hydration states. (A) Calculated free energy barriers at the DFTB2/ff14SB level for each state, with error bars obtained from the standard deviation between the results of three independent umbrella sampling runs. In the snapshots, the acylenzyme state is shown, with the DW in ball and stick (imipenem with orange carbons). Left: hydration state 1 (less hydrated, only one water molecule bonded to KCX@OQ2); DW acts as an H-bond donor to the 6α-hydroxyethyl group. Middle: hydration state 2 (more hydrated, two water molecules bonded to KCX@OQ2); DW acts as a hydrogen donor to the 6α-hydroxyethyl group. Right: hydration state 1; DW acts as hydrogen acceptors to the hydroxyl group. (B) Correlation between experimental free energy barriers (values taken from Figure 2) and calculated free energy barriers obtained from umbrella sampling. The same color scheme as in panel A is used. Correlation is indicated for OXA-48 and -517 with DW acting as H-bond donor and OXA-163 and -405 with DW as H-bond acceptor. (C) Free energy surfaces for imipenem deacylation by OXA-48 under different hydration states and H-bonding patterns. The order is the same as in panel A. Minimum free energy paths (MFEPs) indicated by graph spheres, with AC = acylenzyme, TS = transition state, and TI = tetrahedral intermediate. Similar MFEPs are obtained for the other variants (Figure S4).

For each variant, the highest free energy barrier for deacylation is obtained when KCX is fully hydrated with both carboxylate oxygens accepting hydrogen bonds from two water molecules. This hydration state has an even larger impact on the barrier for the β_5_–β_6_ loop variants than for WT OXA-48 (Figure 3A and Table S2). When only a single water molecule interacts with the carboxylate oxygen that is not involved in proton transfer (KCX@OQ2), the barriers are lower. When the DW *accepts* an H-bond from the imipenem 6α-hydroxyethyl group, the barriers are similar for each variant and always higher than those obtained with DW *donating* an H-bond to the 6α-hydroxyethyl group. In other words, for each variant, the lowest free energy barrier for deacylation is obtained in this situation (DW donating an H-bond to the 6α-hydroxyethyl group, KCX@OQ2 accepting an H-bond from a single water molecule). The different H-bonding patterns and hydration states also change the minimum free energy pathway (MFEP), causing a shift in the transition state location (Figure 3C). In particular, when DW accepts a hydrogen bond from the 6α-hydroxyethyl group, the transition state occurs further along the reaction (closer to the tetrahedral intermediate), especially for the proton transfer reaction coordinate. When DW donates an H-bond but KCX is more hydrated, the overall reaction path is very similar, but the TS still occurs slightly later (which is expected due to a reduction in the KCX proton affinity). These changes in the MFEP are consistent for all four variants studied (Figures S4 and S5), with representative structures of the minima and transition states shown in Figures S6 and S7. Although the order of the predicted energy barriers in the most reactive active site conformation for the four OXA-48-like proteins is consistent with experimental data (10.8 ± 0.8, 11.5 ± 0.7, 11.1 ± 0.9, and 10.9 ± 0.7 kcal mol^–1^ for OXA-48, -163, -405, and -517 respectively), the differences are too small to explain the experimental *k*_cat_ values.^20,37,41,42^ This suggests that the variation in *k*_cat_ values among these variants may be attributed to differences in attaining or maintaining this most reactive conformation (where a single water molecule interacts with KCX@OQ2 and DW donates an H-bond to the 6α-hydroxyethyl group).

For carbapenem hydrolysis by OXA-48 and -163, it has been shown that *k*_cat_ is very close to the deacylation rate; i.e., deacylation is the rate-limiting step.^21^ Owing to the QM method (DFTB2) we used here, our calculated energy barriers for deacylation are consistently underestimated compared to experimental data (Figure 2), as found previously for this method for similar reactions.^62,63^ Our previous work on carbapenem deacylation by OXA-48 demonstrated that DFTB2 underestimates barriers by ∼6.3 kcal mol^–1^ compared to more accurate DFT calculations (M06-2X/def2-TZVP).^22^ This work also established that the difference in the hydrolysis efficiency of imipenem and meropenem by OXA-48 can be attributed to a subtle change in the hydrogen bonding between DW and the 6α-hydroxyethyl group, with DW acting as donor with imipenem but as acceptor with meropenem. Here, if we assume that for both OXA-48 and -517, the DW acts as a hydrogen donor with hydration state 1 during the reaction, the barriers, corrected to DFT level (+6.3 kcal mol^–1^), would be 17.1 and 17.2 kcal mol^–1^, respectively. Further, if we assume that for OXA-163 and -405, DW accepts a hydrogen bond from the 6-hydroxyethyl moiety instead, the corrected barriers would be 19.3 and 18.8 kcal mol^–1^. For all four enzymes, these values are close to those inferred from *k*_cat_ values (Figure 2), and there is an excellent correlation between the calculated and experimental free energy barriers of the four variants (Figure 3B, where *R* ^2^ > 0.99). These results further support that TI formation in deacylation is (primarily) rate-determining for carbapenem hydrolysis by OXA-48-like enzymes. This conclusion is consistent with previous findings for both ceftazidime and carbapenem hydrolysis by these enzymes,^22,28^ as well as carbapenem hydrolysis by class A BLs.^62,63^ In addition, these results indicate that the difference in catalytic rate between OXA-48 and -517, on the one hand, and between OXA-163 and -405, on the other, lies in the orientation (and thus hydrogen bond pattern) of the DW. To understand the origin of this subtle difference, we analyze the conformational dynamics of the respective complexes in the following sections.

### β_5_–β_6_ Loop Mutations Cause Differences in Acylenzyme Dynamics around the Active Site

Molecular dynamics simulations of the acylenzyme states were performed for each OXA-48 variant studied (five independent simulations of 120 ns for each) to investigate if the variants have differences in conformational preferences and flexibility (that may be correlated to H-bonding patterns causing differences in deacylation). In the simulations, the “reactive” orientation of the 6α-hydroxyethyl group was maintained. Combined clustering based on the backbone RMSD (Cα atoms) shows a clear separation in the conformational preference among the variants (Table S3). Two distinct clusters are identified, with the following main differences: (1) the backbone distance between Val120 and Leu158 and (2) the conformation of the β_5_–β_6_ loop (Figure 4A). Cluster 1 is dominant in OXA-48 and -517 and has a larger average distance between the Cα atoms of Val120 and Leu158 (11.1 Å) compared to that observed in cluster 2 (10.4 Å), dominant in the presence of OXA-163 and -405 (Figure S8A). This points to a difference in the width of the water channel formed by the Val120 and Leu158 side chains. When the width of the water channel is measured directly (Figure 4B), it is clear that OXA-48 always samples a wider water channel, whereas a more closed water channel dominates in OXA-405. If and how this change in water channel width effects deacylation efficiency are not immediately clear. On the one hand, a wider channel may lead to a somewhat higher likelihood of the DW being in a catalytically relevant position in OXA-48 and -517, as indicated by the radial distribution function (RDF) of water molecules around the electrophilic carbon of imipenem and the oxygen atom of KCX that receives the proton (Figure S8B, first peaks). However, a reactive DW (based on an “ideal” DW position for deacylation of ≤3.0 Å between DW@O and KCX@OQ1 *and* ≤3.5 Å between DW@O and the electrophilic carbon of imipenem) was observed only slightly more frequently in OXA-48 (Table S4). On the other hand, a wider water channel may lead to a somewhat higher hydration around KCX@OQ2, although the catalytically favorable hydration state 1 is still dominant among all four imipenem acylenzyme complexes (average number of solvent H-bond formed with KCX@OQ2 is 1.45, 1.27, 1.06, and 1.45 for OXA-48, -163, -405, and -517, respectively). Overall, there are some changes in water access to KCX and the electrophilic imipenem between the OXA-48 variants (which were previously suggested based on the more open active site in OXA-163 ^21^), but they are quite subtle and unlikely to lead directly to significant differences in deacylation efficiency.

**Figure 4:**
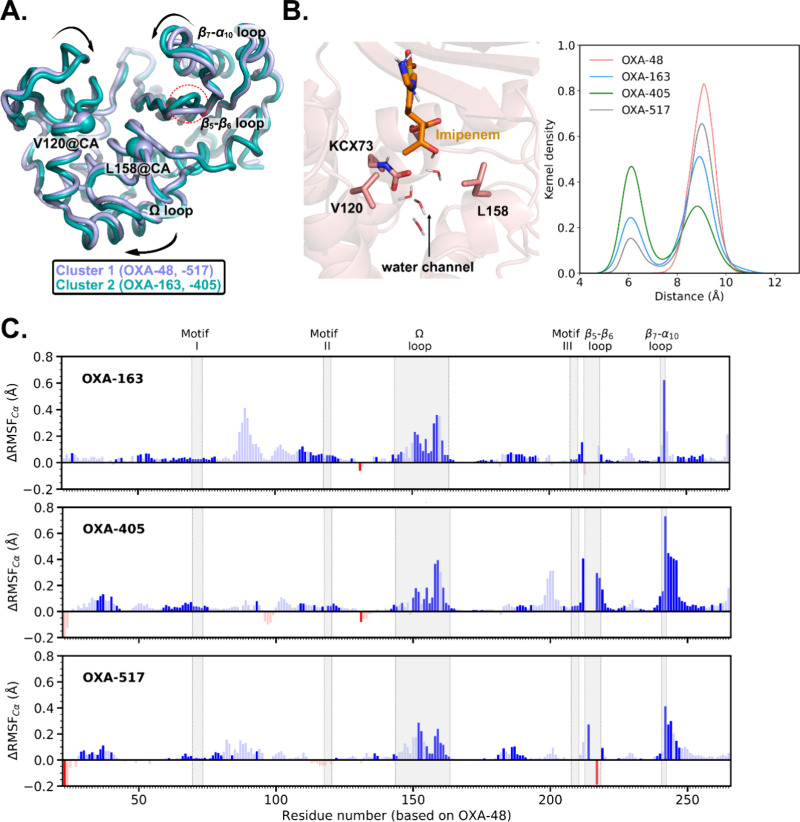
Analysis of imipenem acylenzyme complex dynamics in OXA-48 and the OXA-48 variants. (A) Representative structures of clusters of OXA-48-like proteins based on Cα atoms, with cluster 1 dominant in OXA-48 and -517 and cluster 2 dominant in OXA-163 and -405 (see Table S3). (B) Water channel between Val120 and Leu158 in OXA-48 proteins (left) and distributions of the closest distance between Val120 and Leu158 side chains sampled (right). (C) Change in the flexibility (ΔRMSF_Cα_) of OXA-163, -405, and -517 compared to that of OXA-48. The increase of RMSF_Cα_ is colored in blue; the decrease in red. ΔRMSF_Cα_ values shown in unfaded colors are statistically significant (*p* < 0.05).

The second main difference in the two clusters identified is in the β_5_–β_6_ loop conformation (as indicated based only on β_5_–β_6_ loop residues common to all four variants). This is not surprising given the major changes in this loop. The clear distinction in the β_5_–β_6_ loop backbone conformation between OXA-163 and -405, compared to OXA-48 and -517, is further confirmed by principal component analysis of the common backbone atoms (Figure S9). The four-residue deletions in OXA-163 and -405 change the preferred β_5_–β_6_ loop conformation (cluster 1) such that it is positioned further away from the Ω loop (Figure 4A and Figure S9C). Although OXA-517 also harbors a (two-residue) deletion in the β_5_–β_6_ loop, it still has a similar β_5_–β_6_ loop conformation as that of OXA-48. This difference in positioning is related to the presence or absence of a salt bridge interaction between the β_5_–β_6_ and Ω loops: Arg214 in OXA-48 forms a stable salt bridge with Asp159 in the Ω loop (Figure S10), with Lys214 in OXA-517 occasionally forming an equivalent interaction, whereas no salt bridge is detected for OXA-163 and -405. In turn, this salt bridge affects the flexibility of the Ω loop. All three OXA-48 variants show increased flexibility of the backbone (see ΔRMSF_Cα_ analysis in Figure 4C and Figure S11), with the main increase observed in the Ω loop and OXA-517 having a slightly more stable Ω loop than those of OXA-163 and -405. Another difference in flexibility is observed around the β_7_–α_10_ loop (where there is also a slight difference in the conformation between the identified clusters). The exact reason for this change is not clear, but it may be related to weak interactions with the tail group of imipenem (which is highly flexible in all simulations). Overall, the greater flexibility of the active site loops in OXA-163 and -405 observed here may increase the accessibility of the active site, aiding the hydrolysis of bulkier substrates such as ceftazidime, as suggested previously.^33^ (Notably, in a structure of the ceftazidime acylenzyme in OXA-48 P68A, there is a lack of interpretable electron density for the Ω loop.^34^)

### Active Site H-Bond Networks in OXA-48 Variants Differ due to β_5_–β_6_ Loop Changes

As well as the difference in backbone conformation of the β_5_–β_6_ loop mentioned above, the variants studied here show significant differences in the side chains and their interactions in this loop. In particular, Thr213 is involved in hydrogen bonding networks around the acylated imipenem (Figure 5A). This β_5_–β_6_ loop residue is directly adjacent to the mutation/deletion sites in OXA-163 and -517 and is deleted in OXA-405. Three Thr213 rotamers exist (defined by the χ_1_ dihedral angle, N-Cα-Cβ-OG1): gauche-plus (*g*+) (+60°), trans (*t*) (180°), and gauche-minus (*g*−) (−60° or 300°). In our MD simulations, *g*+ is dominant in all three OXA-48-like enzymes possessing Thr213 (OXA-48, -163, and -517; Figure S12 and Figure 5B). Even though the preferred rotamer is the same in the variants, the interactions with Thr213 can lead to differences in the active site H-bond network. In the OXA-48 imipenem acylenzyme complex, Thr213 predominantly accepts an H-bond from nearby water (switching to H-bonding with the Ser212 carbonyl when the rare *g*– rotamer is sampled) (Figure 5A, left). Due to the two residue deletions, Thr213 in OXA-517 prefers donating a hydrogen bond to a nearby water molecule and does not form an alternative H-bond with Ser212 (Figure 5A, right). For OXA-163, the situation is different: in the preferred *g*+ rotamer, Thr213 predominantly donates an H-bond to the backbone carbonyl of residue 212 (Figure 5A, middle), which is the most commonly found conformation here (Figure 5B).

**Figure 5:**
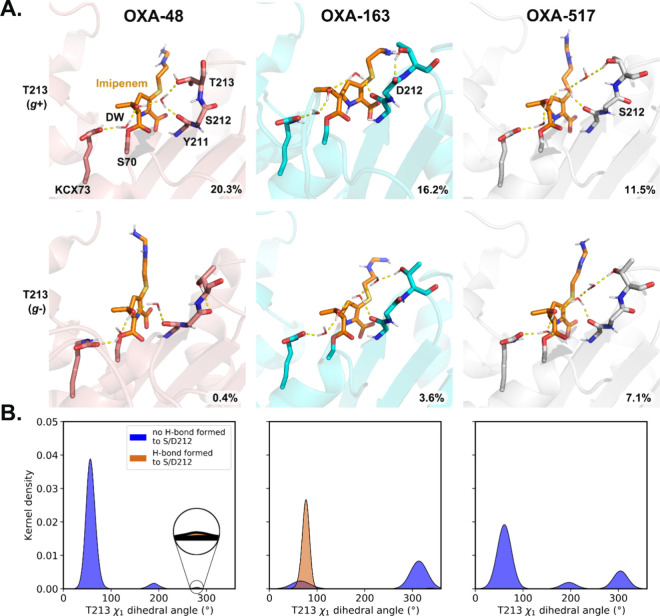
Different H-bond networks among acylenzyme complexes. (A) Representative snapshots of different Thr213 side chains from simulations. Only polar hydrogens are shown. Key residues (pink) and imipenem (orange) are shown as sticks. H-bonds are shown as yellow dash lines. The fraction of the H-bond network is shown in the bottom right corner; these are relatively low due to the strict criteria (donor–acceptor distance less than 3.0 Å and a donor–hydrogen–acceptor angle between ±135° and 180°). (B) H-bond analysis of Thr213 and Ser/Asp212 and analysis of Thr213 side chain dihedral angle show different H-bonding patterns among acylenzyme complexes (all frames are used here).

In OXA-48, Thr213 is part of a stable H-bond network, where both the side chain hydroxyl of Thr213 and the backbone carbonyl of Tyr211 act as hydrogen acceptor to a water molecule. This is consistent with what has been observed crystallographically (OXA-48 K73A imipenem acylenzyme complex, PDB ID: 7KH9, chain B).^21^ This water further accepts an H-bond from the imipenem 6α-hydroxyethyl hydroxyl group, leading to the DW donating (rather than accepting) an H-bond to this imipenem hydroxyl group (Figure 5A, left and Figure S13). Because in OXA-163, Thr213 forms an H-bond with Asp212 instead (consistent with PDB ID: 7KHZ, OXA-163 K73A imipenem acylenzyme complex),^21^ the equivalent water molecule donates H-bonds to both the 6α-hydroxyethyl hydroxyl group and the Tyr211 backbone. This then leads to DW acting as a hydrogen bond acceptor (Figure 5A, middle and Figure S13). For OXA-517, both the Thr213 *g*+ and *g*– rotamers donate an H-bond to a nearby water molecule, which in turn interacts with a second water that donates H-bonds to both the Tyr211 backbone carbonyl and the imipenem 6α-hydroxyethyl hydroxyl group (Figure 5A, right and Figure S13). The latter then also leads to DW accepting (rather than donating) an H-bond to the imipenem hydroxyl group. Due to the Thr213 deletion in OXA-405 and thus the lack of a hydrogen bond donor in this position, a similar H-bond network to OXA-163 is typically observed: 45.2% of the frames have a water molecule bridging the 6α-hydroxyethyl hydroxyl and the Tyr211 carbonyl such that the water donates a hydrogen bond to both. (The conformation of the OXA-405 β_5_–β_6_ loop backbone is very different from the other three OXA-48 variants; see Figure S9.) The Ser212-Pro217 peptide bond of OXA-405 is in the *trans* conformation (Figure S14), whereas the equivalent E216/K214-Pro217 peptide bond in OXA-48 and -517 adopts the more common *cis* isomer.^64^

When only the frames of the acylenzyme MD simulation trajectories are considered that have DW in a “reactive” position (3.0 Å between DW@O and KCX@OQ1 to facilitate proton transfer and 3.5 Å between DW@O and imipenem carbon to facilitate nucleophilic attack), a significant difference in DW H-bonding preference was found (Figure 6A). Specifically, in OXA-48, the DW acts preferentially as a hydrogen donor to the imipenem hydroxyl group, but this is not the case for OXA-163, -405, and -517. As demonstrated above, the DW acting as hydrogen bond donor to the imipenem hydroxyl group favors deacylation. Due to the residue deletion in the β_5_–β_6_ loop, OXA-517 requires one additional water molecule to bridge the H-bonding interaction between the imipenem hydroxyl group and Thr213 (Figure 5A), leading to a preference for the DW acting as an acceptor in the acylenzyme MM MD simulations. To better assess the relative energetic difference between the conformations with DW acting as either a donor or an acceptor to the imipenem hydroxyl group in the OXA-48 variants, we compared QM/MM potential energies of the relevant energy minima, where the hydrogen bond network is treated as QM (Figure 6B and Figure S15). For both OXA-48 and OXA-517, the energy difference is similar and small (2.6 ± 0.6 and 2.2 ± 0.4 kcal mol^–1^, respectively; Table S5), indicating that both hydrogen bonding patterns are easily accessible. In contrast, the energy difference is much larger for OXA-163 and -405 (5.3 ± 0.8 and 7.5 ± 1.0 kcal mol^–1^, respectively; Table S5), indicating that adopting a reactive conformation with DW as the H-bond donor is associated with a significant energy penalty. Overall, these results suggest that in OXA-48 and -517, the water-mediated H-bond network involving Thr213 favors the formation of the DW H-bonding pattern that leads to efficient imipenem hydrolysis.

**Figure 6:**
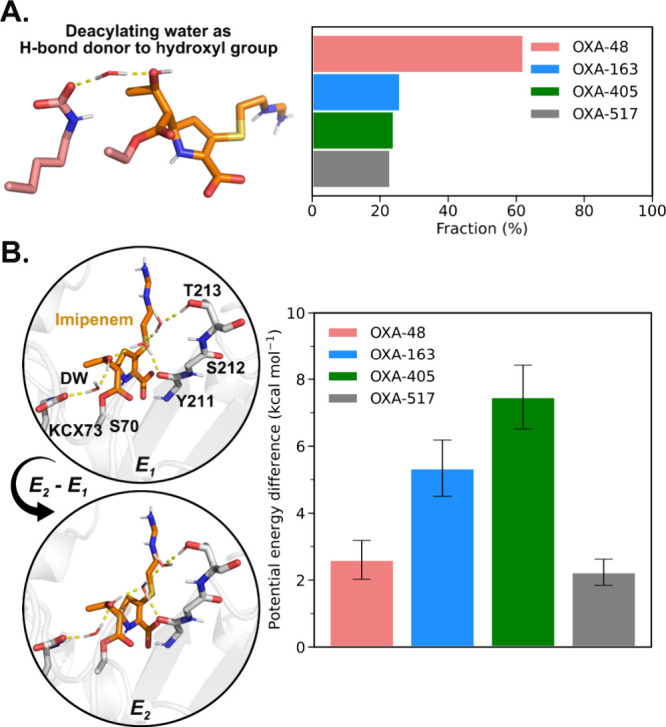
The deacylating water H-bonding pattern that favors imipenem deacylation is energetically preferred in OXA-48 and -517. (A) Analysis of H-bonding patterns between DW and imipenem hydroxyl group of frames with DW in the active site ready for reaction (≤3.0 Å between DW@O and KCX@OQ1 and 3.5 Å between DW@O and the electrophilic carbon). (B) Representative structures at local minima corresponding to different deacylating water H-bonding patterns in OXA-517 (left), used for computing potential energy differences (see Figure S15 for other variants). Only polar hydrogens are shown. Key residues (gray) and imipenem (orange) are shown as sticks, and H-bonds are shown as yellow dash lines. The potential energy differences (*E*_2_ – *E*_1_) for OXA-48 and its variants (right) were calculated at the M06-2X/def2-TZVP//ff14SB level.

### Different Imipenem Binding Pose Leads to Lower Affinity in OXA-517

Although the *k*_cat_ values from the experiment indicate similar deacylation rates for OXA-48 and OXA-517, the overall imipenem deacylation efficiency is expected to be significantly lower in OXA-517 due to a >30-fold difference in *K*_M_ (see Figure 2). To investigate whether noncovalent binding of imipenem contributes to this difference, we conducted MM MD simulations of Michaelis complexes of imipenem with the OXA-48-like proteins. Clustering of all trajectories based on the carbapenem core of imipenem after alignment on the active site (see Methods) shows a subtle, but significant, difference in binding pose between OXA-517 and the other variants (Figure S16): imipenem is shifted toward the β_5_–β_6_ loop.

To estimate if this altered position affects the relative binding affinity, we conducted MM/GBSA analysis on all trajectories. Although this method is approximate, it should be well-suited here for comparison of relative binding energies: the ligand is the same throughout, and the enzyme variants are closely related (i.e., any systematic errors should cancel out). The MM/GBSA calculations indicate that imipenem has a significantly lower binding affinity to OXA-517 than to OXA-48 (*p* < 0.001), whereas the predicted binding energies of imipenem to OXA-48, -163, and -405 are similar (Table 1). Decomposing the MM/GBSA binding affinity by residue indicates that the shift in the position of bound imipenem in OXA-517 weakens the interactions with Ile102/Trp105/Ser118/Thr209 (compared to that of OXA-48), resulting in the lower estimated binding affinity (Figure S17). Due to the differences in the β_5_–β_6_ loop (see above), all three variants further show significantly different contributions of β_5_–β_6_ loop residues to the binding energy, but the less favorable contributions from Thr213 (and Pro217) compared to those from OXA-48 appear to be compensated by preceding residues (Tyr211 and, for OXA-163 and -405, also Thr209 and Lys208). Our analysis indicates no significant change in overall imipenem binding energy between OXA-48 and OXA-163 (*p* = 0.27), consistent with no significant difference in *K*_M_ from a direct comparison of kinetics between these variants.^21^ Others also reported *K*_M_ values for OXA-163 and OXA-405 that indicate no significant difference between them,^37,42^ consistent with the similar binding energies estimated here (*p* = 0.70). The difference in *K*_M_ values for OXA-163 between refs 21 and 37,42 may thus originate from different experimental protocols. Notably, for these enzymes where the deacylation rate is particularly slow, the *K*_M_ may also be influenced by the accumulation of the acylenzyme (potentially due to tautomerisation of the pyrroline ring to the Δ^1^ tautomer, which is less reactive^23^).

**Table 1:** Binding Energy of Imipenem Michaelis Complexes

enzyme	MM/GBSA binding energy (kcal mol^–1^)^a^
OXA-48	–34.9 ± 1.4
OXA-163	–36.0 ± 2.9
OXA-405	–36.4 ± 1.8
OXA-517	–29.9 ± 3.0

a Standard deviations obtained from MM/GBSA calculations on 10 independent 10 ns MD trajectories.

## Conclusions

The inactivation of antibiotics is a major contributor to antimicrobial resistance, with the hydrolysis of β-lactam antibiotics by β-lactamases being a primary mechanism.^4,5,65^ Understanding the molecular basis of β-lactam resistance mediated by β-lactamases should help in anticipating and combating antibiotic resistance. Although the enhanced cephalosporin activity by the OXA-163 and -405 variants (compared to that of the OXA-48) may be ascribed to the increased active site volume and flexibility, allowing larger substrates such as ceftazidime to bind and react efficiently, it has remained unclear why these variants also show a significant reduction in carbapenem hydrolysis. For OXA-163 vs OXA-48, pre-steady-state kinetic studies have shown that this reduction is entirely due to a slower deacylation reaction in OXA-163, but the (atomistic) reasons for this reduction were yet unknown. By conducting detailed simulations of the rate-limiting deacylation reaction, as well as of the acylenzyme complexes, our work here provides such explanations based on comparison of OXA-48 with three OXA-48 variants with changes in the β_5_–β_6_ loop: OXA-163, -405, and -517. First, we show that the hydration around the catalytic carboxylated Lys73 (KCX) and the DW H-bonding pattern significantly influences the energy barrier in all variants, as was previously found for OXA-48.^22^ The hydration of catalytic bases involved in (partially rate-limiting) proton abstraction plays an important role not only in BLs, but also in other enzymes, such as ketosteroid isomerase and triosephosphate isomerase,^66,67^ with reduced hydration of the base lowering the activation energy. Importantly, the barriers for deacylation from QM/MM reaction simulations obtained here agree very well with rates obtained experimentally and *if* OXA-163 and -405 do not adopt the reactive orientation of the DW (donating an H-bond to the carbapenem 6α-hydroxyethyl group). We show that adopting this arrangement in these variants, which lack carbapenemase activity, is indeed associated with an energy penalty and how this is directly related to the changes in the β_5_–β_6_ loop. Specifically, changes to a water-mediated H-bond network between the carbapenem 6α-hydroxyethyl group and Thr213 in the β_5_–β_6_ loop, previously observed in crystal structures of OXA-163 and OXA-48,^21,24^ cause the different preference in DW orientation and H-bonding.

While OXA-517 has been reported to have approximately the same turnover number for imipenem as OXA-48 (indicating efficient imipenem deacylation), its overall efficiency for imipenem breakdown is poor due to a much increased *K*_M_.^41^ Here, we show that this difference is probably related to a lower binding affinity for intact imipenem in OXA-517, in turn due to a subtle shift of the imipenem binding position, which impairs its interactions with the active site. No such differences in noncovalent binding were observed for OXA-163 and -405.

In summary, by using multiscale simulations, we conclude that the higher *k*_cat_ for imipenem breakdown by OXA-48 and -517, compared to those of OXA-163 and -405, can be attributed to the preference for different H-bonding interactions involving DW. Variations in the β_5_–β_6_ loop, mainly involving Thr213, result in distinct H-bond networks around the 6α-hydroxyethyl group of imipenem. This strong water-mediated H-bond network in OXA-48 leads to a preference for the DW H-bonding pattern favored for reaction. In OXA-517, this H-bond network is weakened, but it can still stabilize the H-bonding pattern linked to high activity.

These insights from simulations complement and extend the information available through protein crystallography and kinetic studies. In particular, we provide a detailed explanation of how and why changes in the β_5_–β_6_ loop lead to a reduction in carbapenemase activity, which is something that was not yet understood. Our work thereby highlights how mutations can lead to very subtle effects at the level of individual hydrogen bonds, which in turn lead to significant effects on enzyme activity. We thereby provide a deeper understanding of how mutations around the active site can lead to modifications of the hydrolytic profile of β-lactamases and thus also how such bacterial enzymes may adapt to confer resistance against β-lactam antibiotics. Such knowledge is important because, in general, clinical resistance mechanisms often arise from similarly small, structurally cryptic changes that collectively erode antibiotic efficacy. Furthermore, understanding exactly how loop modifications and (thereby) hydrogen bonding networks can influence β-lactam turnover may create opportunities for further rational design, e.g., new antibiotics that disrupt the key interactions required for efficient deacylation that we identify. Our results add to similar insights obtained for other serine BL-substrate combinations, encompassing both BL-antibiotic^22,44,68^ and BL-inhibitor systems,^69^ and thereby demonstrate how (multiscale) simulations can provide key insights related to understanding, and potentially combating, BL-conferred antibiotic resistance.

## Supplementary Material



## Data Availability

The input files, including the settings and initial structures for all simulations and QM/MM potential energy calculations, as well as the parameters for the carboxylated Lys73 and imipenem in both the free-ligand and Ser70-bound form, are made available at Zenodo: 10.5281/zenodo.18300786.

## Notes

Detailed description of applied restraints and the definition of the QM region used in the reaction simulations; multiple sequence alignment of OXA-48 and its variants; visualization of different imipenem 6α-hydroxyethyl orientations; full results of free energy surfaces for deacylation in different H-bond patterns and hydration states; cluster analysis of acylenzyme and Michaelis complex MM MD simulations; active site water and principal component analysis of acylenzyme MM MD simulations; salt bridge and Thr213 rotamer sampling of acylenzyme MM MD simulations; visualization of structures used in QM/MM potential energy calculations; and energy decomposition of the difference in MM/GBSA binding energies (PDF)

## References

[1] Frieri M., Kumar K., Boutin A. (2017). Antibiotic resistance. J. Infect. Public Health.

[2] Friedman N. D., Temkin E., Carmeli Y. (2016). The negative
impact of antibiotic
resistance. Clin. Microbiol. Infec..

[3] Naghavi M., Vollset S. E., Ikuta K. S., Swetschinski L. R., Gray A. P., Wool E. E., Aguilar G. R., Mestrovic T., Smith G., Han C., Hsu R. L., Collaborators,
G. A. R. (2024). Global burden of
bacterial antimicrobial resistance 1990–2021: a systematic
analysis with forecasts to 2050. Lancet.

[4] Wilke M. S., Lovering A. L., Strynadka N. C. J. (2005). β-lactam
antibiotic resistance:
a current structural perspective. Curr. Opin.
Microbiol..

[5] Narendrakumar L., Chakraborty M., Kumari S., Paul D., Das B. (2023). β-lactam
potentiators to re-sensitize resistant pathogens: discovery, development,
clinical use and the way forward. Front. Microbiol..

[6] Bush K., Bradford P. A. (2016). β-lactams
and β-lactamase inhibitors: an
overview. Csh Perspect. Med..

[7] Sauvage E., Kerff F., Terrak M., Ayala J. A., Charlier P. (2008). The penicillin-binding
proteins: structure and role in peptidoglycan biosynthesis. Fems Microbiol. Rev..

[8] Zapun A., Contreras-Martel C., Vernet T. (2008). Penicillin-binding proteins and beta-lactam
resistance. Fems Microbiol. Rev..

[9] Ambler R. P. (1980). The structure
of beta-lactamases. Philos. Trans. R Soc. Lond
B Biol. Sci..

[10] Tooke C. L., Hinchliffe P., Bragginton E. C., Colenso C. K., Hirvonen V. H. A., Takebayashi Y., Spencer J. (2019). β-lactamases and β-lactamase
inhibitors in the 21st century. J. Mol. Biol..

[11] Brem J., Cain R., Cahill S., McDonough M. A., Clifton I. J., Jiménez-Castellanos J. C., Avison M. B., Spencer J., Fishwick C. W. G., Schofield C. J. (2016). Structural
basis of metallo-β-lactamase, serine-β-lactamase and penicillin-binding
protein inhibition by cyclic boronates. Nat.
Commun..

[12] Pitout J. D. D., Peirano G., Kock M. M., Strydom K. A., Matsumura Y. (2019). The global
ascendency of OXA-48-type carbapenemases. Clin.
Microbiol. Rev..

[13] Evans B. A., Amyes S. G. (2014). OXA beta-lactamases. Clin. Microbiol.
Rev..

[14] Hirvonen V. H., Spencer J., Van Der Kamp M. W. (2021). Antimicrobial resistance conferred
by OXA-48 β-lactamases: towards a detailed mechanistic understanding. Antimicrob. Agents Chemother..

[15] Walther-Rasmussen J., Hoiby N. (2006). OXA-type carbapenemases. J. Antimicrob. Chemother..

[16] Chen Y., Bonnet R., Shoichet B. K. (2007). The acylation
mechanism of CTX-M
β-lactamase at 0.88 Å resolution. J. Am. Chem. Soc..

[17] Hermann J. C., Ridder L., Mulholland A. J., Höltje H. D. (2003). Identification
of Glu166 as the general base in the acylation reaction of class A
β-lactamases through QM/MM modeling. J.
Am. Chem. Soc..

[18] Hermann J. C., Ridder L., Höltje H. D., Mulholland A. J. (2006). Molecular
mechanisms of antibiotic resistance: QM/MM modelling of deacylation
in a class A beta-lactamase. Org. Biomol. Chem..

[19] Golemi D., Maveyraud L., Vakulenko S., Samama J. P., Mobashery S. (2001). Critical involvement
of a carbamylated lysine in catalytic function of class D β-lactamases. P Natl. Acad. Sci. USA.

[20] Docquier J. D., Calderone V., De Luca F., Benvenuti M., Giuliani F., Bellucci L., Tafi A., Nordmann P., Botta M., Rossolini G. M. (2009). Crystal structure of
the OXA-48 beta-lactamase reveals mechanistic diversity among class
D carbapenemases. Chem. Biol..

[21] Stojanoski V., Hu L. Y., Sankaran B., Wang F., Tao P., Prasad B. V. V., Palzkill T. (2021). Mechanistic
basis of OXA-48-like
β-Lactamases’ hydrolysis of carbapenems. ACS Infect. Dis..

[22] Hirvonen V. H. A., Weizmann T. M., Mulholland A. J., Spencer J., van der
Kamp M. W. (2022). Multiscale simulations identify origins of differential
carbapenem hydrolysis by the OXA-48 beta-lactamase. ACS Catal..

[23] Tooke C. L., Hinchliffe P., Beer M., Zinovjev K., Colenso C. K., Schofield C. J., Mulholland A. J., Spencer J. (2023). Tautomer-Specific Deacylation
and Ω-Loop Flexibility Explain the Carbapenem-Hydrolyzing Broad-Spectrum
Activity of the KPC-2 β-Lactamase. J.
Am. Chem. Soc..

[24] Smith C. A., Stewart N. K., Toth M., Vakulenko S. B. (2019). Structural
insights into the mechanism of carbapenemase activity of the OXA-48
β-lactamase. Antimicrob. Agents Chemother..

[25] Akhtar A., Pemberton O. A., Chen Y. (2020). Structural Basis for Substrate Specificity
and Carbapenemase Activity of OXA-48 Class D β-Lactamase. ACS Infect. Dis..

[26] Papp-Wallace K. M., Kumar V., Zeiser E. T., Becka S. A., van den
Akker F. (2019). Structural Analysis of The OXA-48 Carbapenemase Bound to A “Poor”
Carbapenem Substrate, Doripenem. Antibiotics.

[27] Fonseca F., Chudyk E. I., van der
Kamp M. W., Correia A., Mulholland A. J., Spencer J. (2012). The Basis for Carbapenem Hydrolysis by Class A β-Lactamases:
A Combined Investigation using Crystallography and Simulations. J. Am. Chem. Soc..

[28] Lohans C. T., Freeman E. I., Groesen E. V., Tooke C. L., Hinchliffe P., Spencer J., Brem J., Schofield C. J. (2019). Mechanistic
insights into β-lactamase-catalysed carbapenem degradation through
product characterisation. Sci. Rep..

[29] Toth M., Smith C. A., Antunes N. T., Stewart N. K., Maltz L., Vakulenko S. B. (2017). The role of conserved surface hydrophobic
residues
in the carbapenemase activity of the class D beta-lactamases. Biol. Crystallogr..

[30] Vercheval L., Bauvois C., di Paolo A., Borel F., Ferrer J.-L., Sauvage E., Matagne A., Frère J.-M., Charlier P., Galleni M. (2010). Three factors that modulate
the activity of class D β-lactamases and interfere with the
post-translational carboxylation of Lys70. Biochem.
J..

[31] Paetzel M., Danel F., de Castro L., Mosimann S. C., Page M. G. P., Strynadka N. C. J. (2000). Crystal structure of the class D
β-lactamase OXA-10. Nat. Struct. Biol..

[32] Chiou J., Cheng Q., Shum P. T., Wong M. H., Chan E. W., Chen S. (2021). Structural and functional
characterization of OXA-48: insight into
mechanism and structural basis of substrate recognition and specificity. Int. J. Mol. Sci..

[33] Fröhlich C., Gama J. A., Harms K., Hirvonen V. H., Lund B. A., van der Kamp M. W., Johnsen P. J., Samuelsen Ø., Leiros H. K. S., Bradford P. A. (2021). Cryptic β-Lactamase evolution
is driven by low β-lactam concentrations. Msphere.

[34] Fröhlich C., Sørum V., Thomassen A. M., Johnsen P. J., Leiros H. K. S., Samuelsen Ø., Gales A. C. (2019). OXA-48-mediated
ceftazidime-avibactam resistance is associated with evolutionary trade-offs. Msphere.

[35] Salamonsen D., Pierangelini A., Buda K., Wang D., van der
Kamp M. W., Tokuriki N., Adrian Bunzel H., Fro̷hlich C. (2025). Evolution tunes functional sub-state interconversion
to boost enzyme function. bioRxiv.

[36] Poirel L., Castanheira M., Carrer A., Rodriguez C. P., Jones R. N., Smayevsky J., Nordmann P. (2011). OXA-163, an OXA-48-related
class D beta-lactamase with extended activity toward expanded-spectrum
cephalosporins. Antimicrob. Agents Chemother..

[37] Oueslati S., Nordmann P., Poirel L. (2015). Heterogeneous hydrolytic features
for OXA-48-like beta-lactamases. J. Antimicrob.
Chemother..

[38] Dabos L., Zavala A., Bonnin R. A., Beckstein O., Retailleau P., Iorga B. I., Naas T. (2020). Substrate specificity
of OXA-48 after β5-β6 loop replacement. ACS Infect. Dis..

[39] Lund B. A., Thomassen A. M., Carlsen T. J. O., Leiros H. K. S. (2017). Structure, activity
and thermostability investigations of OXA-163, OXA-181 and OXA-245
using biochemical analysis, crystal structures and differential scanning
calorimetry analysis. Acta Crystallogr. F.

[40] Oueslati S., Retailleau P., Marchini L., Berthault C., Dortet L., Bonnin R. A., Iorga B. I., Naas T. (2020). Role of arginine
214 in the substrate specificity of OXA-48. Antimicrob. Agents Chemother..

[41] Dabos L., Raczynska J. E., Bogaerts P., Zavala A., Girlich D., Bonnin R. A., Dortet L., Peyrat A., Retailleau P., Iorga B. I. (2023). Structural and biochemical features of OXA-517:
a carbapenem and expanded-spectrum cephalosporin hydrolyzing OXA-48
variant. Antimicrob. Agents Chemother..

[42] Oueslati S., Retailleau P., Marchini L., Dortet L., Bonnin R. A., Iorga B. I., Naas T. (2020). Biochemical and structural characterization
of OXA-405, an OXA-48 variant with extended-spectrum beta-lactamase
activity. Microorganisms.

[43] Garcia-Viloca M., Gao J., Karplus M., Truhlar D. G. (2004). How enzymes work: analysis by modern
rate theory and computer simulations. Science.

[44] Hirvonen V. H. A., Mulholland A. J., Spencer J., van der
Kamp M. W. (2020). Small Changes in Hydration Determine Cephalosporinase
Activity of OXA-48 β-Lactamases. ACS Catal..

[45] Vanquelef E., Simon S., Marquant G., Garcia E., Klimerak G., Delepine J. C., Cieplak P., Dupradeau F. Y. R. (2011). E. D.
Server: a web service for deriving RESP and ESP charges and building
force field libraries for new molecules and molecular fragments. Nucleic Acids Res..

[46] Jakalian A., Jack D. B., Bayly C. I. (2002). Fast, efficient generation of high-quality
atomic charges. AM1-BCC model: II. Parameterization and validation. J. Comput. Chem..

[47] Wang J. M., Wolf R. M., Caldwell J. W., Kollman P. A., Case D. A. (2004). Development
and testing of a general amber force field. J. Comput. Chem..

[48] Olsson M. H., Sondergaard C. R., Rostkowski M., Jensen J. H. (2011). PROPKA3: consistent
treatment of internal and surface residues in empirical pKa predictions. J. Chem. Theory Comput..

[49] Case, D. A. ; Belfon, K. ; Ben-Shalom, I. Y. ; Brozell, S. R. ; Cerutti, D. S. ; Cheatham, T. E. III ; Cruzeiro, V. W. D. ; Darden, T. A. ; Duke, R. E. ; Giambasu, G. AMBER 2020; University of California: San Francisco, 2020.

[50] Maier J. A., Martinez C., Kasavajhala K., Wickstrom L., Hauser K. E., Simmerling C. (2015). ff14SB: improving
the accuracy of
protein side chain and backbone parameters from ff99SB. J. Chem. Theory Comput..

[51] Jorgensen W. L., Chandrasekhar J., Madura J. D., Impey R. W., Klein M. L. (1983). Comparison
of simple potential functions for simulating liquid water. J. Chem. Phys..

[52] Akhter S., Lund B. A., Ismael A., Langer M., Isaksson J., Christopeit T., Leiros H.-K. S., Bayer A. (2018). A focused fragment
library targeting the antibiotic resistance enzyme - Oxacillinase-48:
Synthesis, structural evaluation and inhibitor design. Eur. J. Med. Chem..

[53] Schneider K. D., Karpen M. E., Bonomo R. A., Leonard D. A., Powers R. A. (2009). The 1.4
Å Crystal Structure of the Class D β-Lactamase OXA-1 Complexed
with Doripenem. Biochemistry.

[54] Roe D. R., Cheatham T. E. (2013). PTRAJ and CPPTRAJ:
software for processing and analysis
of molecular dynamics trajectory data. J. Chem.
Theory Comput..

[55] Elstner M. (2006). The SCC-DFTB
method and its application to biological systems. Theor. Chem. Acc..

[56] Kumar S., Bouzida D., Swendsen R. H., Kollman P. A., Rosenberg J. M. (1992). The weighted
histogram analysis method for free-energy calculations on biomolecules
0.1. The method. J. Comput. Chem..

[57] Marcos-Alcalde I., Setoain J., Mendieta-Moreno J. I., Mendieta J., Gómez-Puertas P. (2015). MEPSA: minimum
energy pathway analysis for energy landscapes. Bioinformatics.

[58] Liu D. C., Nocedal J. (1989). On the limited memory
BFGS method for large scale optimization. Math
Program.

[59] Neese F. (2025). Software update:
The ORCA program system—Version 6.0. WIREs Comput. Mol. Sci..

[60] Miller B. R., McGee T. D., Swails J. M., Homeyer N., Gohlke H., Roitberg A. E. (2012). MMPBSA.py: an efficient
program for end-state free
energy calculations. J. Chem. Theory Comput..

[61] Onufriev A., Bashford D., Case D. A. (2004). Exploring
protein native states and
large-scale conformational changes with a modified generalized born
model. Proteins.

[62] Hirvonen V. H. A., Hammond K., Chudyk E. I., Limb M. A. L., Spencer J., Mulholland A. J., van der Kamp M. W. (2019). An Efficient Computational Assay
for β-Lactam Antibiotic Breakdown by Class A β-Lactamases. J. Chem. Inf. Model.

[63] Chudyk E. I., Limb M. A. L., Jones C., Spencer J., van der
Kamp M. W., Mulholland A. J. (2014). QM/MM simulations as an assay for
carbapenemase activity in class A β-lactamases. Chem. Commun..

[64] MacArthur M. W., Thornton J. M. (1991). Influence of Proline
Residues on Protein Conformation. J. Mol. Biol..

[65] Darby E. M., Trampari E., Siasat P., Gaya M. S., Alav I., Webber M. A., Blair J. M. A. (2023). Molecular mechanisms of antibiotic
resistance revisited. Nat. Rev. Microbiol..

[66] van
der Kamp M. W., Chaudret R., Mulholland A. J. (2013). QM/MM modelling
of ketosteroid isomerase reactivity indicates that active site closure
is integral to catalysis. FEBS J..

[67] Liao Q., Kulkarni Y., Sengupta U., Petrović D., Mulholland A. J., van der Kamp M. W., Strodel B., Kamerlin S. C. L. (2018). Loop
Motion in Triosephosphate Isomerase Is Not a Simple Open and Shut
Case. J. Am. Chem. Soc..

[68] Lima A. H., van der Kamp M. W. (2025). Insights
into the Enhanced Ceftazidime Hydrolysis by
Ent385 AmpC β-Lactamase from Multiscale Simulations. ACS Catal..

[69] Fritz R. A., Alzate-Morales J. H., Spencer J., Mulholland A. J., van der Kamp M. W. (2018). Multiscale
Simulations of Clavulanate Inhibition Identify
the Reactive Complex in Class A β-Lactamases and Predict the
Efficiency of Inhibition. Biochemistry.

